# Binding Properties and Stability of the Ras-Association Domain of Rap1-GTP Interacting Adapter Molecule (RIAM)

**DOI:** 10.1371/journal.pone.0031955

**Published:** 2012-04-16

**Authors:** Heikki Takala, Jari Ylänne

**Affiliations:** Department of Biological and Environmental Science and Nanoscience Center, University of Jyväskylä, Jyväskylä, Finland; BioScience Project, United States of America

## Abstract

The Rap1-GTP interacting adapter protein (RIAM) is an important protein in Rap1-mediated integrin activation. By binding to both Rap1 GTPase and talin, RIAM recruits talin to the cell membrane, thus facilitating talin-dependent integrin activation. In this article, we studied the role of the RIAM Ras-association (RA) and pleckstrin-homology (PH) domains in the interaction with Rap1. We found that the RA domain was sufficient for GTP-dependent interaction with Rap1B, and the addition of the PH domain did not change the binding affinity. We also detected GTP-independent interaction of Rap1B with the N-terminus of RIAM. In addition, we found that the PH domain stabilized the RA domain both *in vitro* and in cells.

## Introduction

Integrins are transmembrane adhesion receptors that are important in cell adhesion and migration. These heterodimers contribute to vital events, such as development, haemostasis and immunity [1]. Integrins are bidirectional signaling receptors; they mediate signals both from the inside of the cell to the outside and vice versa. Integrin inside-out signaling can be triggered by several transmembrane receptors, and the generated signal is propagated in the cytoplasm to the integrin cytoplasmic domains. The cytoplasmic interactions then cause changes in integrin affinity for extracellular ligands, thus activating integrins [2]. The Ras-family of small GTPases are important signaling elements that control integrin function [3]. The small GTPases act as molecular switches by cycling between an active GTP-bound and an inactive GDP-bound conformation. The cycle is facilitated by guanine nucleotide exchange factors (GEFs) and GTPase-activating proteins (GAPs) [4], [5]. Whereas Ras GTPases are mainly thought to play a role in cell proliferation and cell survival, Rap1 has a pronounced role in integrin-dependent cell adhesion and spreading [4]. It is shown to regulate the affinity and avidity of integrins in leukocytes [6]–[8] and in platelets [9] as well as in epithelial cells [10].

**Figure 1:**
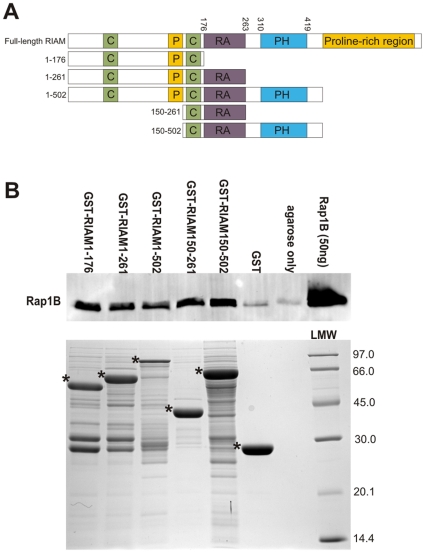
The binding of active Rap1B to RIAM constructs. (**A**) Schematic diagram of the RIAM constructs. RIAM constructs of varying lengths were expressed and tested; the residue range of each construct is shown on the left. Full-length RIAM is included in the figure, with domain boundaries marked according to UniProtKB sequence annotation of the entry AB1IP_HUMAN and [14]. Abbreviations: C – coiled-coil, P – proline-rich region, RA – Ras-association domain, PH – pleckstrin-homology domain. (**B**) Pull-down of purified Rap1B-*Gpp(NH)p* with GST-RIAM constructs and controls. The GST-RIAM constructs indicated in panel *A* were bound to glutathione Sepharose, and soluble 1 µM purified Rap1B was allowed to bind. The Western blot membranes were labeled with anti-Rap1B. The 50 ng Rap1B input is included as a reference. The amount and size of GST-RIAM constructs used in the assay (asterisks) are indicated in a Coomassie Brilliant Blue-stained SDS-PAGE gel. The molecular weight marker (LMW, GE Healthcare) with corresponding molecular weights are indicated on the right.

Canonical Rap1 effectors bind to the conserved switch region of GTPase with their Ras-associating (RA) domain or similar Ras-binding domain (RBD) [5]. These domains form a well-defined ubiquitin-like fold that interacts with the GTPase in order to form an inter-protein β sheet between the proteins [11]. This interaction is GTP-sensitive because of the stabilizing role of GTP in switch regions [5], [12]. One of the Rap1 effectors [13], called the Rap1-GTP interacting adapter molecule (RIAM) has been shown to link Rap1 signaling to β1, β2 and β3 integrin activation [14]–[17]. The key event in intergrin activation is the binding of talin to the cytoplasmic domain of integrins [18]. This event is facilitated by RIAM, which, by binding both Rap1 and talin, recruits the integrin activation complex to the plasma membrane. At the plasma membrane, talin can then interact with and activate integrins [15]–[17]. RIAM belongs to the Mig-10/RIAM/Lamellipodin (MRL) family of adaptor proteins [14]. The MRL members share a similar domain composition, with consecutive Ras-association (RA) and pleckstrin-homology (PH) domains flanked by proline-rich regions. This central RA-PH domain pair is preceded by a conserved patch of 27 residues that is predicted to form a coiled-coil structure (Figure 1A). The related Grb7 family of adaptor proteins shares several structural characteristics with MRL proteins, including the RA-PH domain pair [14].

In addition to talin and Rap1, RIAM has been shown to bind to the profilin and Ena/VASP family of proteins with its proline-rich regions. Therefore, it is shown to be an important mediator in actin cytoskeleton dynamics and is involved in lamellipodia formation [14], [19]. RIAM has also been shown to interact with the ADAP/SKAP-55 protein module, which has a recruiting role regarding the RIAM/Rap1 complex [20]. The interaction involves the central region of RIAM, including the RA-PH domain pair. Both RA and PH domains of RIAM have also been shown to be important in the interaction with Rap1. In yeast two-hybrid assays, the interaction required an intact RA-PH domain pair [14], whereas other experiments have questioned the requirement of the PH domain for the interaction [17]. Therefore, the exact role of the PH domain in Rap1-RIAM interaction has remained partially elusive.

To study the role of the RA and PH domains in the interaction of RIAM with Rap1, we used pull-down assays in combination with thermal stability and proteolysis assays. We show that the RA domain of RIAM was sufficient for interaction with Rap1B *in vitro*. Furthermore, the RA domain required a consecutive PH domain in order to remain stable *in vitro* and in cells. Therefore, our results propose a novel stabilizing role of the PH domain in MRL proteins.

## Results

### RIAM Binds to Rap1B with its N-terminus and the RA Domain

Rap1-GTP interacting adapter molecule (RIAM), as its name indicates, interacts with small GTPase Rap1 [14]. Previous studies have indicated that the RA domain is required for the interaction [14], [17]. To find out whether the RA domain is sufficient for Rap1 binding, pull-down assays of purified active Rap1B-*Gpp(NH)p* with different GST-RIAM constructs were conducted. The two Rap1 isoforms (Rap1A and Rap1B) are 95% identical in amino acid sequence. The difference is mainly restricted to the C-terminus of both isoforms that is excluded from the Rap1B construct used in this article (amino acids 1–167). Therefore the following results should be applicable to both Rap1 isoforms. The RIAM constructs consisted of an N-terminal fragment (RIAM1-176), the RA domain (RIAM1-261, RIAM150-261) or both RA and PH domains (RIAM1-502, RIAM150-502) (Figure 1A). All the RIAM constructs interacted with Rap1B (Figure 1B).

### RIAM Binds Rap1B with a Similar Affinity as Other Ras–effector Interactions. This Interaction is Dependent on Rap1B Activity State

**Figure 2:**
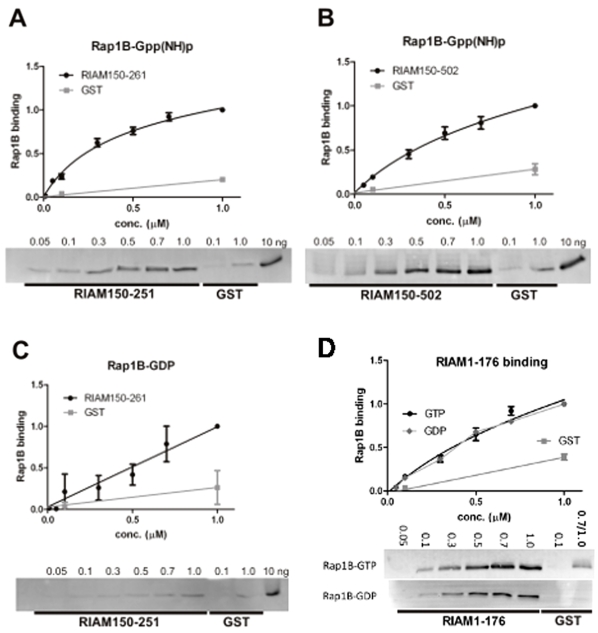
Quantitative binding assays of Rap1B to RIAM fragments. Binding assays indicate specific GTP-dependent interaction between Rap1B and GST-RIAM-RAconstructs. (**A** and **C**) RIAM150-261 binds to active Rap1B-*Gpp(NH)p* in a specific manner (K_d_ 0.33±0.09 µM), whereas the binding is lost in Rap1B-GDP (Kd not determined). (**B**) RIAM150-502 binds Rap1B-*Gpp(NH)p* with an affinity (K_d_ 0.7±0.4 µM) similar to that of RIAM150-261. (**D**) RIAM 1-176 binds identically to Rap1B-*Gpp(NH)p* (GTP) and Rap1B-GDP. In all panels the binding of GST-RIAM binding to purified Rap1B in 0.05, 0.1, 0.3, 0.5, 0.7 and 1.0 µM concentrations is shown as well as the unspecific binding of GST control. The 10 ng Rap1B load is shown for comparison. The binding was quantified by labeling membranes with anti-Rap1 after Western blotting, and it is expressed as Rap1B binding. The graph shows total binding (*black* curve) and the unspecific background binding (*grey* line). The dissociation constant was calculated as the ratio of specific to unspecific binding, normalized to maximal Rap1B binding in each experiment (mean ± S.E. (*error bars*); n ≥ 4, exept for D.

In order to further characterize the interactions, the assays we repeated with various concentrations of either Rap1B-*Gpp(NH)p* or Rap1B-GDP. The binding of Rap1B-GDP to the RIAM RA domain construct (RIAM150-261) was remarkably weaker than that of Rap1B-GTP (Figure 2B and C). This shows that the binding of Rap1B to the RIAM RA domain is GTP dependent. On the other hand, the binding of Rap1B to the N-terminal fragment of RIAM (RIAM1-176) was not GTP dependent (Figure 2D). The fragment containing the RA and PH domains (RIAM150-502), bound Rap1B-*Gpp(NH)p* similarly as the RA-domain construct. The dissociation constants (K_d_) were (0.33 ± 0.09) µM for RIAM150-261 and (0.7 ± 0.4) µM for RIAM150-502 (Figure 2A and B). The residuals fitted well to the curve determined by nonlinear regression, and the values for the goodness of the fit of both RIAM150-261 (R^2^: 0.96) and RIAM150-502 (R^2^: 0.92) were close to 1.0. The similar affinities of these two constructs imply that the PH domain does not have a clear impact on the binding of RIAM to Rap1B *in vitro*.

The dissociation constants of RIAM1-176 with Rap1B-*Gpp(NH)p* or Rap1B-GDP, or RIAM150-261 with Rap1B-GDP could not be determined as the saturation could not be achieved. Taken together, our binding measurements suggest that the main Rap1 GTP-dependent binding site resides on the RA domain of RIAM. This is consistent with a GTP-dependent interaction of Rap1 switch regions with the RA domain [21]. In addition to this the N-terminus of RIAM also participates in the interaction, independent on the GTP binding of Rap1.

### The RIAM RA Domain is Unstructured, but Stabilized by the PH Domain Both *in vitro* and *in vivo*

**Figure 3:**
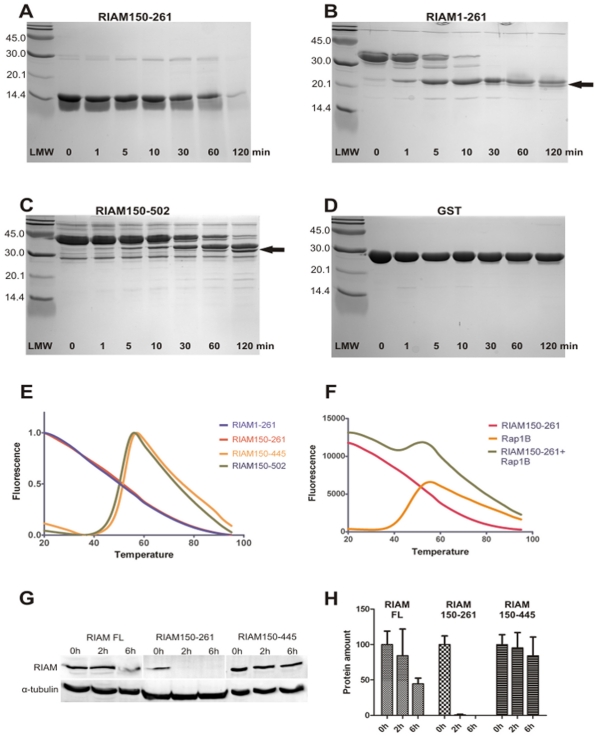
Limited proteolysis, thermal stability and in-cell proteolysis assays indicate an unstable RA domain, but a stable RA-PH domain pair. (**A-D**) The limited proteolysis analysis of RIAM150-502 (*A*), RIAM1-261 (*B*), RIAM150-261 (*C*) and GST (*D*). Fragments that appeared upon α-chymotrypsin treatment are marked with *arrows*. The incubation times are included in the figure. (*E*) Temperature denaturation profiles of RIAM1-261 (*blue*), RIAM150-261 (*red*), RIAM 150-445 (*yellow*) and RIAM150-502 (*green*). (**F**) The profiles of Rap1B-*Gpp(NH)p* (*yellow*) and RIAM150-261 (*red*) in individual measurements and measured together (*green*). All measurements were repeated 5 times and the mean graph is shown. The fluorescence values are normalized in *E* and plotted as arbitrary units in *F*. (**G**) *In vivo* proteolysis assay of RIAM constructs in CHO cells. Tranfected myc-RIAM constructs after various cycloheximide treatment times (0 h, 2 h, 6 h) are labeled with anti-myc. In order to check the comparable sample amount, the samples were also labeled for α-tubulin. (**H**) The graph shows qualitatively the same changes to the myc-RIAM amount after cycloheximide treatment (mean ± SEM (*error bars*); n  =  2).

Although RIAM-Rap1B interaction does not require a PH domain *in vitro*, the RIAM PH domain could have an impact on RA domain stability. The crystal structure of Grb10, a relative of the MRL proteins, reveals a compact RA-PH structure [22]. As the RA and PH domains are also in close contact with each other in MRL proteins, we next tested whether the PH domain would affect the stability of the RA domain [14], [22]. To study this, we first employed limited proteolysis. RIAM constructs were digested with chymotrypsin and run in SDS-PAGE after different incubation times (Figure 3A-D). All the RIAM fragments (Figure 3A-C) were considerably less stable than glutathione S-transferase (GST) (Figure 3D) in this analysis. No partial digestion products were observed from the RIAM150-261 construct (Figure 3A). The RIAM1-261 construct gave a partially stable subfragment migrating at Mw ∼22 kDa (Figure 3B) and the RIAM150-502 gave a subfragment migrating at Mw ∼35 kDa (Figure 3C), based on the SDS-PAGE analysis. Further peptide fingerprinting (Figure S1) suggested that the subfragment of RIAM1-261 was truncated from the N-terminus and thus contained the RA domain. From the subfragments of RIAM150-502, peptides ranging over amino acids 182–459 could be identified, suggesting the presence of both RA and PH domains in this partially stable subfragment. Thus, based on limited proteolysis, the predicted RA domain and the RA-PH domain pair could be identified as folded units.

To further investigate the stability of the RIAM constructs, the ThermoFluor method [23] was used. ThermoFluor distinguishes the folded and unfolded nature of a protein by using a fluorophore that binds to the hydrophobic parts of protein. When the protein is thermally denatured, more of the hydrophobic interior will be exposed and the fluorophore emission increases. The ThermoFluor profiles of RIAM1-261 and RIAM150-261 were featureless (Figure 3E), suggesting that the constructs have unfolded characteristics. This result differs from the limited proteolysis assay, but it is possible that the small amount of partially stable RA domain seen in limited proteolysis may not be detectable in the ThermoFluor assay or that this domain contains hydrophopic surface accessible for the fluorophore. In contrast to this, RIAM150-445 gave a characteristic denaturation curve with a melting temperature (T_m_) of 52.5°C, and the RIAM150-502 constructs had a T_m_ of 50.0°C (Figure 3E). A possible explanation for the observed difference between the RA domain and the RA-PH domain fragment is that the PH domain of RIAM is required for stability of the RA domain.

Also, the possibility that Rap1B binding could stabilize the RIAM RA domain was tested. The proteins RIAM150-261 and Rap1B-*Gpp(NH)p* were analyzed with ThermoFluor individually or together in a 1:1 molar ratio. The concentration of each protein was 10 µM, which was far above the concentration required for the saturation of the interaction, as determined above. The profile of RIAM150-261 was not affected when Rap1B-*Gpp(NH)p* (T_m_ 46°C) was included (Figure 3F). The resulting fluorescence was the sum of the fluorescence values of the individual proteins, but no clear change in the RIAM150-261 fluorescence input was seen. Therefore, Rap1B binding did not affect the stability of the RA domain in the ThermoFluor assay.

To test whether the PH domain is also required for the stability of the RA domain *in vivo,* CHO cells were transfected with myc-tagged RIAM constructs having full-length RIAM (RIAM FL), the RA domain only (RIAM150-261) or the RA-PH domain pair (RIAM150-445). The transfected cells were treated with cycloheximide to inhibit protein synthesis, and the samples were collected after various incubation times to observe protein turnover. The RA domain-only construct was almost totally degraded after 2 hours, whereas the full-length RIAM and the RA-PH construct were partially stable even after 6 hours (Figure 3G and H). In addition, the RA-PH construct appeared even more stable than the full-length RIAM construct, which underlines the stable characteristics of the domain pair. This result further confirms that RIAM requires both the RA and PH domains in order to form a stable entity *in vivo*.

## Discussion

In the present study, we characterized the direct binding of small GTPase Rap1B to its effector RIAM and the effect of the PH domain for RIAM stability. With a biochemical interaction assay, we showed that RIAM interacts with Rap1B primarily via its RA domain *in vitro*. The interaction between RIAM RA domain and Rap1B had a similar K_d_ as other Ras-related proteins had with their effectors that range between 20 nM and 2 µM [24]. This interaction was GTP-dependent, because Rap1B-GDP showed only background binding. This implies that the interaction forms between the Rap1B switch regions and RIAM RA domain, as with other Rap1B effector interactions [5], [11]. The GTP stabilizes the switch regions, thus enabling effector binding; GDP relaxes these effector binding regions, thus abrogating binding [5], [12]. In addition to this, we also showed that the N-terminus of RIAM participates in Rap1 binding in a GTP independent manner.

Our results are consistent with Lee and others, who showed that RIAM1-301, lacking the PH domain, was sufficient for interaction with Rap1 for integrin activation [17]. The N-terminal interaction observed here also fits with previous results showing that the N-terminal part of RIAM enhances the overall interaction with Rap1 [14], but is not sufficient for activation of integrins [17]. On the other hand, in a yeast two-hybrid assay, both the RA and PH domains of RIAM were required in order to interact with active Rap1 [14]. This discrepancy may be explained by our finding that the PH domain is required for stabilization of RIAM RA domain both *in vitro* and in cell culture. It is therefore possible that the activity of the partially unstable RA domain may be detected in some experimental conditions but not in others.

PH domains usually bind to phosphoinositides, and they have been shown to be important for the membrane localization of MRL proteins and the closely-related Grb7 family. Lamellipodin, a homologous MRL protein, cannot bind to Rap1 with its RA domain, but its PH domain binds to PtdIns(3,4)P_2_. The PH domain localizes lamellipodin to the plasma membrane [25]. When Grb14 was mutated in order to abrogate the phosphoinositide binding of its PH domain [14], it lost its membrane localization; Grb14 showed reduced interaction with Ras GTPase and failed to mediate normal insulin signaling [22]. This infers that the membrane association via the PH domain can be important for the interaction with small GTPases. The PH membrane association would position the RA-PH unit on the cell membrane in such a way that the RA domain could optimally bind to small GTPases [22].

In contrast to lamellipodin and Grb7 proteins, no phosphoinositides have been characterized as binding to RIAM so far, although close homology with lamellipodin suggests that RIAM might have similar specificity [25]. On the other hand, the RIAM PH domain has been implicated in adaptor protein interaction. In T-cells, the RIAM PH domain is required for ADAP/SKAP-55 complex interaction [20]. The RA-PH tandem domain pair is not sufficient for membrane targeting itself. Instead, interaction with SKAP-55 is required [20]. RIAM binding to SKAP-55 does not compete with Rap1, and this binding requires both the RA and (especially) PH domains of RIAM. Abrogation of RIAM/SKAP-55 interaction leads to impaired cell adhesion after TCR activation [20].

The MRL proteins and Grb7 proteins have a characteristically conserved RA-PH domain pair [14]. The crystal structure of Grb10 shows a pair of canonically folded RA and PH domains that have an extensive interface (1326 Å^2^) between the domains [22]. The interface has a hydrophobic cluster that is also conserved in the MRL protein family members; the corresponding residues are usually hydrophilic in the single RA domains of other proteins [22]. This provides a possible structural explanation for the requirement of the PH domain for RA domain stability reported here. Furthermore, in the Grb10 RA-PH structure, the G-protein binding surface of the RA domain is located opposite of the PH domain interaction surface [22]. This is consistent with our finding that the RIAM RA domain alone is sufficient for Rap1 interaction.

## Materials and Methods

### Antibodies

The c-Myc antibody (9E10) was from Santa Cruz Biotechnology (Santa Cruz, CA). The polyclonal Rap1 antibody, polyclonal anti-mouse HRP-conjugate and polyclonal anti-rabbit HRP-conjugate were from Millipore (Temecula, CA). The α-tubulin antibody (DM1A) was from Cedarlane (Ontario, Canada).

### Cloning and Constructs

The plasmid containing full-length RIAM [17] was a kind gift from Dr. Mark Ginsberg. RIAM fragments were amplified by standard PCR and introduced to a modified pGEX-4T3 vector (GE Healthcare) with a tobacco etch virus protease cleavage site. For assays in cells, full-length RIAM was replaced with truncated inserts with ClaI and XbaI restriction sites. Construct pGEX-4T3/Rap1B was generously provided by Dr. Alfred Wittinghofer.

### Protein Expression and Purification

For protein expression, BL21 GOLD cells were transformed, and positive clones were selected with 100 µg/ml ampicillin. Protein expression was induced with 0.4 mM IPTG at OD 0.6, and the expression was conducted at 23°C for 18 h. After expression, cells were collected by centrifugation and re-suspended in PBS. Cells were disrupted by French Press (2000 psi) and the lysate was centrifuged at 20,000 × g for 30 min. The soluble part of the lysate was coupled to Glutathione Sepharose 4B (GE Healthcare Life Sciences) by incubating for 2 h at +4°C and washed with PBS. The bound GST-fusion protein was eluted with reduced glutathione (Sigma). The protein was further purified with size exclusion chromatography using a HiLoad 16/600 Superdex 75 column (GE Healthcare Life Sciences) in 100 mM NaCl, 1 mM DTT, 20 mM Tris pH 8.0. The selected fractions were concentrated and flash-frozen for later use.

### Rap1B Nucleotide Exchange Reaction

Before size exclusion chromatography, Rap1B was activated with 100x the molar amount of GDP or non-hydrolysable GTP-analog *Gpp(NH)p* (Sigma), as in [26]. The reaction was conducted in 40 mM Tris, 12 mM EDTA, 3 mM DTT pH 7.4 for 1 h at 23°C, and it was stopped by adding 25 mM MgCl_2_. Rap1B was then purified with size exclusion chromatography as above, but 3 mM MgCl_2_ was included in the buffer.

### Pull-down Experiments and Affinity Assays

The pull-down experiments were performed in Buffer A (50 mM Tris pH 7.5, 200 mM NaCl, 2 mM MgCl_2_, 10% glycerol, 1 mM DTT, 1% Triton X-100). The desired amount of purified Rap1B was incubated for 1 h in 10 µl of GST-RIAM-coupled Glutathione Sepharose 4B (GE Healthcare Life Sciences). The samples were then washed 5 times with Buffer A (centrifugations at 1700 × g for 2 min RT) and eluted with SDS electrophoresis sample buffer.

### Immunoblotting and Densitometric Analysis

The samples from the pull-down assays were run on an SDS-PAGE and the fractionated samples were analyzed by Western blotting and detected with Millipore (Temecula, CA) HRP-conjugated secondary antibodies and enhanced chemiluminescence (ECL) Western blotting substrate (product number 32106; Pierce, Rockford, IL). The intensities of the protein bands were analyzed with ImageJ (http://rsbweb.nih.gov/ij/). The affinity constants were estimated as in [27] by using the GraphPad Prism 5.03 program (GraphPad Software, Inc, La Jolla, CA).

### Limited Proteolysis

RIAM constructs were analyzed by limited proteolysis [28] with α-chymotrypsin (Sigma), as in [29]. Protease was added to the protein in a 1:1000 ratio, and proteolysis reactions were performed in 20 mM Tris, pH 8.0, 100 mM NaCl, 1 mM DTT at room temperature. Samples were taken after various incubation time intervals and analyzed in 12% SDS-polyacrylamide gel electrophoresis (PAGE) with Coomassie protein staining. The migration of the proteolytic fragments was analyzed with the Quantity One 4.6.3 (Bio-Rad) program.

### Peptide Mass Fingerprinting

After SDS-PAGE fractionation, Coomassie stained gel bands were excised from the gel and digested with trypsin. The tryptic peptides were analyzed by Bruker UltrafleXtreme MALDI-ToF mass spectrometer (Bruker Corporation, Billerica, MA) and correlated with the protein sequence to estimate the protein area subjected to chymotrypsin digestion as described elsewhere [30].

### Thermofluor Experiments

The thermal stability of the proteins was determined using a C1000 Thermal cycler and CFx96 Real-Time system (Bio-Rad). Thermal denaturation over a 20°C – 95°C temperature gradient was monitored in 0.5°C/30 s increments. Samples consisted of 5 µM or 10 µM protein and 5x SYPRO Orange fluorescent dye (Invitrogen) in 25 µl final volume of 100 mM NaCl, 1 mM DTT, 20 mM Tris pH 8.0. For measurements with Rap1B-*Gpp(NH)p*, MgCl_2_ was included. Figures from the experiments were made with GraphPad Prism 5.03.

### Cell Culture and Proteolysis Assay

Chinese hamster ovary (CHO) cells were cultured according to normal practice in a medium consisting of 10% fetal bovine serum, 1% non-essential amino acids (GIBCO) and desired antibiotics. Cells were transfected with Lipofectamine 2000 (Invitrogen), according to the manufacturer’s instructions. 24 h after transfection, 500 µg/ml cycloheximide (Sigma) was added to the culture medium and incubated for 2 h or 6 h. 0 h samples were not treated with Cycloheximide. After incubation periods, the cells were harvested, lysed into Buffer A and analyzed by Western blotting. Figures from the experiments were made with ImageJ and GraphPad Prism 5.03.

## Supporting Information

Figure S1 **Analysis of the tryptic peptides and correlation with the protein sequence.** The results of mass spectrometric analysis of tryptic peptides from the fragments generated by limited proteolysis (arrows in Figure 3B and C) are shown for the RIAM1-261, spanning from the N-terminus to the end of the RA domain, and RIAM150-502, including the RA-PH domain pair. In each panel, the upper box shows the identified peptides under the input sequence. The lower print lists the observed and calculated peptide masses and sequences. Note that the sequence numbering in the upper box starts from the beginning of the construct. Both constructs have a vector-derived sequence GAMG at the N-terminus.
(PDF)
