# Supplementary material for: Binding Properties and Stability of the Ras-Association Domain of Rap1-GTP Interacting Adapter Molecule (RIAM)
Source: PLoS One. 2012 Apr 16;7(4):e31955. doi: 10.1371/journal.pone.0031955 (PMC3327698; doi:10.1371/journal.pone.0031955)
Supplement: Figure S1 — Analysis of the tryptic peptides and correlation with the protein sequence. The results of mass spectrometric analysis of tryptic peptides from the fragments generated by limited proteolysis (arrows in Figure 3B and C) are shown for the RIAM1-261, spanning from the N-terminus to the end of the RA domain, and RIAM150-502, including the RA-PH domain pair. In each panel, the upper box shows the identified peptides under the input sequence. The lower print lists the observed and calculated peptide masses and sequences. Note that the sequence numbering in the upper box starts from the beginning of the construct. Both constructs have a vector-derived sequence GAMG at the N-terminus. (PDF) [file pone.0031955.s001.pdf]

A

Sequence data:

riam 1-261

Intensity Coverage:	46.2 % (80764 cnts)	Sequence Coverage MS:	24.0%
Sequence Coverage MS/MS:	0.0%	pI (isoelectric point):	4.1

```
        10         20         30         40         50         60         70         80         90        100        110
GAMGESSEDI DQMFSTLLGE MDLLTQSLGV DTLPPPDPNP PRAEFNYSVG FKDLNESLNA LEDQDLDALM ADLVADISEA EQRTIQAQKE SLQNQHHSAS LQASIFSGAA
       120        130        140        150        160        170        180        190        200        210        220
SLGYGTNVAA TGISQYEDDL PPPPADPVLD LPLPPPPPEP LSQEEEEAQA KADKIKLALE KLKEAKVKKL VVKVHMNDNS TKSLMVDERQ LARDVLDNLF EKTHCDCNVD
       230        240        250        260        270
WCLYEIYPEL QIERFFEDHE NVVEVLSDWT RDTENKILFL EKE
```

riam 1-261
MW:29503.480

GAMGESSEDIDQMFSTLLGEMDLLTQSLGVDTLPPPDPNPPRAEFNYSVGFKDLNESLNALEDQDLDALMADLVADISEAEQRTIQAQKESLQNQHHSASLQASIFSGAASLGYGTNVAATGISQYEDDLPPPP
ADPVLDLPLPPPPPEPLSQEEEEAQAKADKIKLALEKLKEAKVKKLVVKVHMNDNSTKSLMVDERQLARDVLDNLFEKTHCDCNVDWCLYEIYPELQIERFFEDHENVVEVLSDWTRDTENKILFLEKE

Mass Search Matches
Search Parameter: MS Tol.:150.000000 ppm, MSMS Tol.:0.500000 Da, Trypsin Specific cleavages only Sequence Editor
Modifications: Optional: Oxidation (M)

Tree hierarchy	Meas. M/z	Calc. MH+	Int.	Dev. (Da)	Dev. (ppm)	Range	P	Sequence
peak 4	849.395	849.413	18088.978	−0.019	−22.634	193 − 199	0	SLMVDER
peak 5	865.394	865.408	15564.253	−0.015	−17.488	193 − 199	0	SLMVDER 3: Oxidation (M)
peak 8	1045.551	1045.473	4277.279	0.077	73.872	184 − 192	0	VHMNDNSTK
peak 9	1061.465	1061.468	818.171	−0.004	−3.544	184 − 192	0	VHMNDNSTK 3: Oxidation (M)
peak 11	1092.561	1092.557	25145.949	0.003	2.849	204 − 212	0	DVLDNLFEK
peak 23	1317.698	1317.694	5150.648	0.003	2.226	193 − 203	1	SLMVDERQLAR
peak 27	1333.702	1333.689	6696.586	0.012	9.016	193 − 203	1	SLMVDERQLAR 3: Oxidation (M)
peak 29	1478.787	1478.774	4353.589	0.013	8.509	252 − 263	1	DTENKILFLEKE
peak 30	1560.842	1560.838	478.614	0.003	2.080	200 − 212	1	QLARDVLDNLFEK
peak 69	2913.256	2913.264	190.352	−0.009	−3.017	213 − 234	0	THCDCNVDWCLYEIYPELQIER 3: Carbamidomethyl (C) 5: Carbamidomethyl (C) 10: Carbamidomethyl (C)

B

Sequence data:

RIAM150-502

Intensity Coverage:	59.1 % (146360 cnts)	Sequence Coverage MS:	46.8%
Sequence Coverage MS/MS:	0.0%	pI (isoelectric point):	9.3

```
        10         20         30         40         50         60         70         80         90        100        110
GAMGSQEEEE AQAKADKIKL ALEKLKEAKV KKLVVKVHMN DNSTKSLMVD ERQLARDVLD NLFEKTHCDC NVDWCLYEIY PELQIERFFE DHENVVEVLS DWTRDTENKI
       120        130        140        150        160        170        180        190        200        210        220
LFLEKEEKYA VFKNPQNFYL DNRGKKESKE TNEKMNAKNK ESLLEESFCG TSIIVPELEG ALYLKEDGKK SWKRRYFLLR ASGIYYVPKG KTKTSRDLAC FIQFENVNIY
       230        240        250        260        270        280        290        300        310        320        330
YGTQHKMKYK APTDYCFVLK HPQIQKESQY IKYLCCDDTR TLNQWVMGIR IAKYGKTLYD NYQRAVAKAG LASRWTNLGT VNAAAPAQPS TGPKTGTTQP NGQIPQATHS
       340        350        360
VSAVLQEAQR HAETSKDKKP ALGNHHD
```

MW:41439.911

GAMGSQEEEEAQAKADKIKLALEKLKEAKVKKLVVKVHMNDNSTKSLMVDERQLARDVLDNLFEKTHCDCNVDWCLYEIYPELQIERFFEDHENVVEVLSDWTRDTENKILFLEKEEKYAVFKNPQNFYLDNRG
KKESKETNEKMNAKNKESLLEESFCGTSIIVPELEGALYLKEDGKKSWKRRYFLLRASGIYYVPKGKTKTSRDLACFIQFENVNIYYGTQHKMKYKAPTDYCFVLKHPQIQKESQYIKYLCCDDTRTLNQWVMG
IRIAKYGKTLYDNYQRAVAKAGLASRWTNLGTVNAAAPAQPSTGPKTGTTQPNGQIPQATHSVSAVLQEAQRHAETSKDKKPALGNHHD

Mass Search Matches
Search Parameter: MS Tol.: 50.000000 ppm , MSMS Tol.: 0.600000 Da, Trypsin Specific cleavages only Sequence Editor
Modifications: Optional: Oxidation (M)

Tree hierarchy	Meas. M/z	Calc. MH+	Int.	Dev. (Da)	Dev. (ppm)	Range	P	Sequence
peak 1	711.386	711.419	12287.710	−0.034	−47.221	186 − 190	0	YFLLR
peak 2	762.454	762.476	179.642	−0.023	−29.846	110 − 115	0	ILFLEK
peak 7	849.396	849.413	198.565	−0.018	−21.456	46 − 52	0	SLMVDER
peak 9	867.506	867.520	9502.426	−0.015	−16.915	185 − 190	1	RYFLLR
peak 10	997.530	997.535	609.639	−0.006	−6.053	191 − 199	0	ASGIYYVPK
peak 20	1072.495	1072.506	42404.080	−0.011	−9.821	277 − 284	0	TLYDNYQR
peak 21	1092.543	1092.557	676.713	−0.015	−13.641	57 − 65	0	DVLDNLFEK
peak 22	1102.414	1102.429	3242.324	−0.016	−14.462	253 − 260	0	YLCCDDTR 3: Carbamidomethyl (C) 4: Carbamidomethyl (C)
peak 25	1148.640	1148.656	224.069	−0.017	−14.705	110 − 118	1	ILFLEKEEK
peak 36	1217.630	1217.646	7207.119	−0.017	−13.695	261 − 270	0	TLNQWVMGIR
peak 39	1233.627	1233.641	9240.332	−0.015	−11.826	261 − 270	0	TLNQWVMGIR 7: Oxidation (M)
peak 44	1280.590	1280.602	53551.049	−0.013	−9.807	124 − 133	0	NPQNFYLDNR
peak 50	1420.655	1420.686	171.798	−0.031	−22.032	274 − 284	1	YGKTLYDNYQR
peak 54	1504.730	1504.750	2910.046	−0.021	−14.090	229 − 240	1	YKAPTDYCFVLK 8: Carbamidomethyl (C)
peak 67	1907.790	1907.859	799.815	−0.068	−35.823	37 − 52	1	VHMNDNSTKSLMVDER 3: Oxidation (M) 12: Oxidation (M)
peak 95	2460.131	2460.165	1950.580	−0.035	−14.225	207 − 226	0	DLACFIQFENVNIYYGTQHK 4: Carbamidomethyl (C)
peak 97	2797.357	2797.422	112.739	−0.065	−23.356	151 − 175	0	ESLLEESFCGTSIIVPELEGALYLK 9: Carbamidomethyl (C)
peak 101	2913.216	2913.264	1091.337	−0.049	−16.752	66 − 87	0	THCDCNVDWCLYEIYPELQIER 3: Carbamidomethyl (C) 5: Carbamidomethyl (C) 10: Carbamidomethyl (C)
